# Remote Patient Monitoring in Adults Receiving Transfusion or Infusion for Hematological Disorders Using the VitalPatch and accelerateIQ Monitoring System: Quantitative Feasibility Study

**DOI:** 10.2196/15103

**Published:** 2019-12-02

**Authors:** Rik Paulus Bernardus Tonino, Karen Larimer, Okke Eissen, Martin Roelof Schipperus

**Affiliations:** 1 Haga Teaching Hospital The Hague Netherlands; 2 Transfusie- en Transplantatiereacties in Patiënten Leiden Netherlands; 3 Leiden University Medical Center Leiden Netherlands; 4 physIQ, Inc Chicago, IL United States; 5 University Medical Center Groningen Groningen Netherlands

**Keywords:** infusions, intravenous, erythrocyte transfusion, transfusion reaction, wearable electronic devices, telemedicine

## Abstract

**Background:**

Frequent vital sign monitoring during and after transfusion of blood products and certain chemotherapies or immunotherapies is critical for detecting infusion reactions and treatment management in patients. Currently, patients return home with instructions to contact the clinic if they feel unwell. Continuous monitoring of vital signs for hematological patients treated with immunotherapy or chemotherapy or receiving blood transfusions using wearable electronic biosensors during and post treatment may improve the safety of these treatments and make remote data collection in an outpatient care setting possible.

**Objective:**

This study aimed to evaluate patient experiences with the VitalPatch wearable sensor (VitalConnect) and to evaluate the usability of data generated by the physIQ accelerateIQ monitoring system for the investigator and nurse.

**Methods:**

A total of 12 patients with hematological disorders receiving red blood cell transfusions, an intravenous (IV) proteasome inhibitor, or an IV immunotherapy agent were included in the study and wore the VitalPatch for 12 days. Patients completed questionnaires focusing on wearability and nurses completed questionnaires focusing on the usability of the VitalPatch.

**Results:**

A total of 12 patients were enrolled over 9 months, with 4 receiving red blood cell transfusions, 4 receiving IV proteasome inhibitors, and 4 receiving IV immunotherapy. These patients were treated for diseases such as multiple myeloma, myelodysplastic syndrome, and non-Hodgkin lymphoma. Of these patients, 83% (10/12) were aged 60 years and older. A total of 4 patients (4/12, 33%) withdrew from the study (3 because of skin irritation and 1 because of patch connection issues). Patients wore biosensor patches at baseline and for 1-week post administration. Patient-reported outcomes (PROs) were collected at baseline, day 1, day 5, and day 8. No difference in the PRO was observed when nurses or patients applied the patch. PRO data indicated minimal impact on the patient’s life. Ease of use, influence on sleep, impact on follow-up of health, or discomfort with continuous monitoring did not change between baseline and day 8. Changes in PRO were observed on day 5, where a 20% (2/10) increase in skin irritation was reported. Withdrawals because of skin irritation were reported in all cases when wearing the second patch. Nurses reported the placement of the VitalPatch to be easy and felt measurements to be reliable.

**Conclusions:**

Generally, the VitalPatch was well tolerated and shown to be an attractive device because of its wearability and low impact on daily activities in patients, therefore making it suitable for implementation in future studies.

## Introduction

### Background

Although continuous patient monitoring is often thought to be reserved for intensive care units, the need for frequent assessment of vital signs is necessary in other clinical circumstances as well. Examples of these circumstances include during and after transfusion of blood products, and during and after transfusion of certain chemotherapies and immunotherapies [1,2]. Receiving these transfusions and infusions can result in untoward reactions that typically manifest as abnormal vital signs before or simultaneously with an adverse event (AE), also known as an infusion reaction [3-5]. For example, 2.1% of all recipients of blood products experience a transfusion reaction, some of which can be life threatening. In addition, administration of many anticancer drugs has a risk for infusion reactions [6]. Therefore, frequent vital sign monitoring during and after treatment is essential to prevent poor patient outcomes.

Even when intensive vital sign monitoring occurs, infusion reactions can go unrecognized. One reason may be inconsistent vital sign assessment [5,7-10]. In one study, researchers found 27.4% (168/614) of nursing staff estimated respiration rate rather than measure it [11]. In many cases, physicians sent patients home after transfusion and infusion therapy with instructions to contact the clinic if the patient became unwell [6]. This leaves the possibility of late detection of infusion reactions open. These findings demonstrate that strategies for patient assessment during and following transfusion and infusion are suboptimal.

To improve the detection of infusion reactions, various interventions have been explored, such as additional training on identifying possible infusion-associated AEs for nurses [2]. Increasing nurses’ knowledge of risk factors for infusion-associated AEs [12] has been implemented, in addition to guidelines to intensify monitoring of infusion recipients at higher risk. Process changes, such as standardized handoff forms [1] and clinical decision support systems [13], are other strategies used to improve safety of transfusions and infusions. Nonetheless, the challenge of adequate assessment during transfusion and infusions remains.

**Figure 1:**
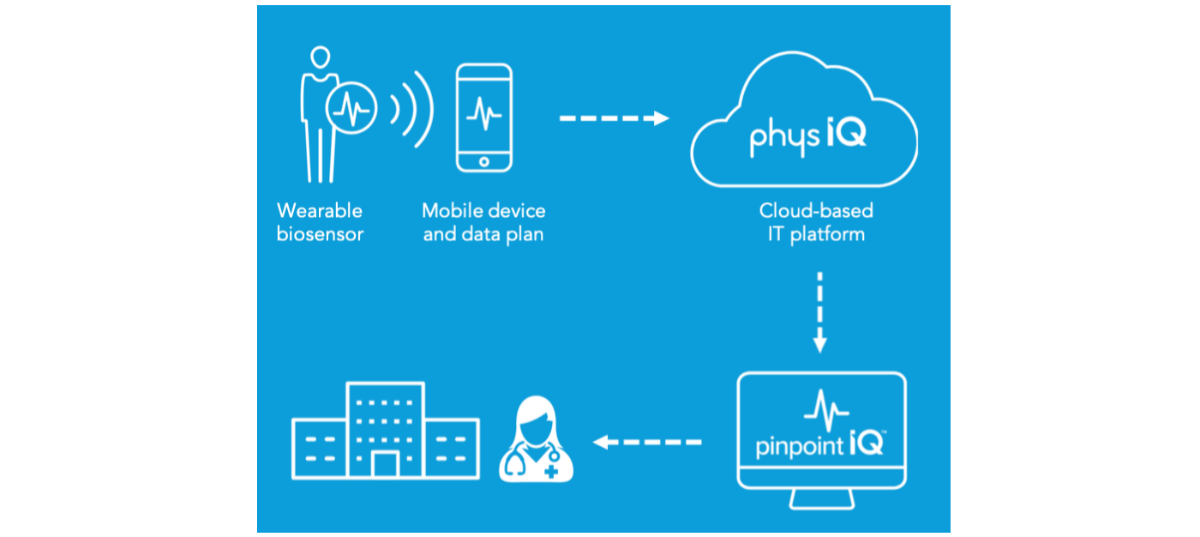
The accelerateIQ end-to-end solution. IT: information technology.

The evolution of digital health and biosensors has opened the possibility of an easier and more effective way of monitoring and analyzing vital signs in patients during and after the receipt of transfusions and infusions . Using these technologies, a health care professional may have greater insight into a patient’s health status. One such product is the accelerateIQ and VitalPatch system. AccelerateIQ is an end-to-end clinical-grade system that collects data from wearable sensors (in this case VitalPatch). AccelerateIQ applies Food and Drug Administration–cleared artificial intelligence analytics to display indicators and data streams through a Web portal. Clinicians can then see any physiological changes indicative of clinical deterioration in patients outside of the acute setting (see Figure 1).

### Objectives

The aim of this study was to evaluate whether the accelerateIQ and VitalPatch system is a practical way to monitor transfusion patients and whether patients find it acceptable to do so. We performed a pilot study to determine whether it was feasible to implement this system in the clinic and to support future studies, with a focus on wearability, usability of data, and safety. These pilot data will be used to inform a further definitive trial to optimize recruitment, treatment compliance, and follow-up protocols.

## Methods

### Study Design

This study was a three-arm, parallel, single center, observational, nonrandomized, open-label feasibility study. The study aimed to explore wearability, usability, and safety of use of the accelerateIQ and VitalPatch system in a population of transfusion and infusion patients.

The medical ethics committee, Zuidwest Holland, granted ethical approval, and the board of the Haga Teaching Hospital granted approval. The trial was performed in accordance with Good Clinical Practice guidelines and the Declaration of Helsinki. Patient data were anonymized to ensure patient privacy. Storage and handling of personal data complied with the General Data Protection Regulation and Medical Treatment Agreement Act, Dutch law.

The study population was comprised of 12 adult participants with a confirmed hematological disorder distributed over 3 different groups. Groups consisted of: (1) 4 patients receiving red blood cell transfusions; (2) 4 patients receiving intravenous (IV) proteasome inhibitors; and (3) 4 patients receiving IV immunotherapy. Patients with severe pulmonary comorbidities, arrhythmias, or other significant conductivity disorders, or with known skin allergies or conditions that might compromise the patient’s safety or quality of data, were excluded. Patients were recruited by their own physician in the Haga Teaching Hospital and included after informed consent was obtained.

### Intervention

Participants in the study received standard care in addition to vital sign monitoring through the accelerateIQ and VitalPatch system. This system consists of the VitalPatch, a disposable adhesive patch biosensor that incorporates 2 surface electrodes with hydrogel and a thermistor on the bottom of the patch. A 4-day battery and an electronic module with an embedded processor, a microelectromechanical system tri-axial accelerometer, and a Bluetooth low-energy transceiver are also part of the sensor. The patch’s sensors facilitate continuous, near real-time monitoring of heart rate, R-R interval, heart rate variability, respiratory rate, single-lead electrocardiography, skin temperature, body posture, fall detection, and activity. Data are sent via Bluetooth to a mobile phone (Samsung J327V, Android), which uploads the data over mobile data networks to the physIQ accelerateIQ cloud platform.

A universal smartphone was provided to maintain quality systems and standards in clinical and trial use. When the phone to patch distance exceeds blue tooth range, which is about 20 feet, the patch will store data and offload it when the phone is back in range. Raw data are then analyzed to extract further vital sign features and to detect vital sign anomalies in the patient physiological response. After that, the smartphone transmits data to the accelerateIQ data platform. physIQ operates in line with national standards for the privacy of health information and regulations for electronic records and signatures, pursuant to the Health Insurance Portability and Accountability Act of 1996 and the Code of Federal Regulations (CFR), respectively.

**Figure 2:**
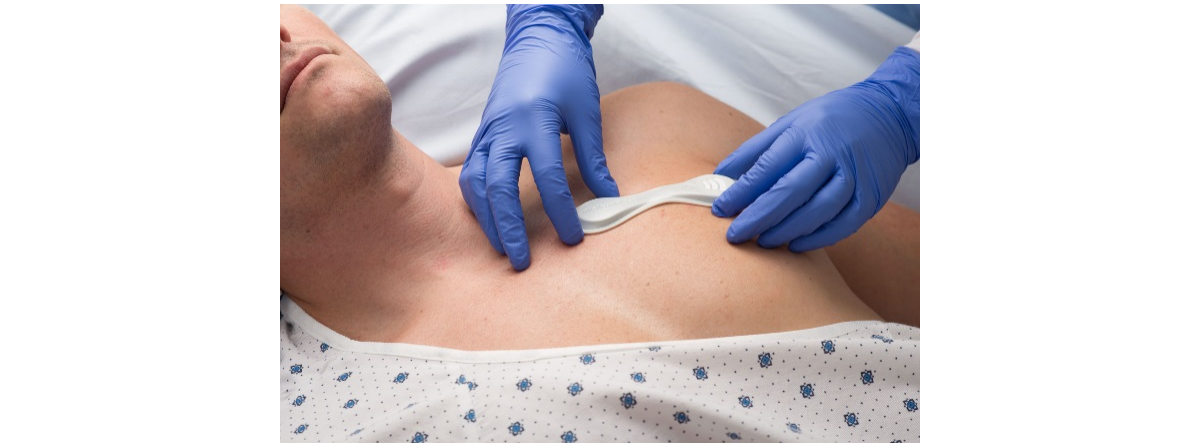
VitalPatch placement.

Patients wore a VitalPatch for a maximum of 12 days. The first patch was placed the week before the start of treatment and worn for 4 days to generate baseline data. The second patch was placed at the start of treatment (or the day before for transfusion patients), and a third patch, necessary because of battery life, was placed after 4 days by the patient at home (see Figure 2). The patch cannot be submerged in water, so while wearing the patch, patients could not swim or bathe. Showering was allowed.

A total of 2 nurses accessed accelerateIQ through a standard Web browser that displayed a patient dashboard. Within the dashboard, the nurses had access to data and analytics to determine if there were any abnormal reactions or potentially dangerous AEs associated with the treatment. The nurses also generally assessed the accuracy of monitoring but did not treat subjects based on portal data.

### Outcome Measures and Data Collection

To understand the acceptability of use of the accelerateIQ plus VitalPatch system by patients and health care professionals, we assessed wearability and usability. Wearability was measured by a questionnaire, completed by patients at baseline, day 1, day 5, and day 8. The questionnaire was in the participant’s native language of Dutch. Questions focused on the patient’s evaluation of their experiences with the VitalPatch. They completed 10 questions by indicating on a Likert-type scale (except for question 2, which was binary [0/1]) whether they agreed (10) or disagreed (1) with the statement. There was one yes/no question.

Participating nurses completed a survey at the end of the study, evaluating their experience with the VitalPatch as a health care professional. The survey consisted of 3 questions with a Likert-type scale and 3 open-ended questions. Both questionnaires were developed by the investigator and underwent face validity. Safety outcomes were also assessed by tracking AEs related to wearing the biosensor.

### Statistical Analysis

No sample size calculation was performed as this was an exploratory study. However, data were reviewed for trends. Descriptive statistics were used and means compared. AEs were analyzed by describing timing and extent of skin reaction.

## Results

### Inclusion

From February to October 2018, 12 patients were enrolled in the study. Participants included 4 patients receiving red blood cell transfusions, 4 receiving proteasome inhibitors, and 4 receiving IV immunotherapy. These patients were receiving treatment for various diseases, including multiple myeloma, myelodysplastic syndrome, and non-Hodgkin lymphoma. A total of 83% (10/12) of the patients were aged 60 years and older, 83% (10/12) were male, and 92% (11/12) were non-Hispanic white (see Table 1). During the study, no abnormal reactions or AEs to transfusions and infusions occurred.

**Table 1:** Baseline characteristics.

Characteristics		Value, n (%)
**Age (years)**		
	40-49	2 (17)
	60-69	6 (50)
	70-79	4 (33)
**Hematological disorder**		
	Multiple myeloma	4 (33)
	Myelodysplastic syndrome	2 (17)
	β-Thalassemia	1 (8)
	Non-Hodgkin lymphoma	5 (42)
**Treatment**		
	Red blood cell transfusion	4 (33)
	R-CHOP^a^	4 (33)
	Carfilzomib and Dexamethason	3 (25)
	Carfilzomib, Lenalidomide and Dexamethasone	1 (8)
**Skin color**		
	White	11 (92)
	Dark	1 (8)
**Sex**		
	Male	10 (83)
	Female	2 (17)

^a^R-CHOP: doxorubicin, cyclophosphamide, vincristine, rituximab, and prednisone.

### Wearability

Wearability was measured through patients completing an 11-question survey (see Table 2 and Multimedia Appendix 1). No difference in patient-reported outcomes (PROs) was observed when either the nurses or the patients applied the patch. Patients considered wearing the patch pleasant (mean 6.7/10), and the patch remained in place (mean 8.1/10). Patients reported little discomfort (mean 2.2/10) and little trouble with sleeping because of the patch (1.6/10). Patients did not feel the patch was in the way (mean 2.3/10) and did not experience restrictions in daily activities because of wearing the patch (mean 1.3/10).

**Table 2:** Questions on patients’ experience with the VitalPatch. Patients responded on a Likert-type scale whether they agreed (10) or disagreed (1) with the statement.

#	Question	Baseline (n=10)	Day 1 (n=12)	Day 5 (n=10)	Day 8 (n=8)	Total (N=40)
1	Was it easy placing the patch? (mean; scale 1-10)	9	9	7.8	8.5	8.6
2	Problems placing patch? (cumulative; yes=1, no=0)	1	1	2	1	1.3
3	Is it pleasant wearing the patch? (mean; scale 1-10)	7.1	6.2	6.1	7.5	6.7
4	Sense that health is being monitored? (mean; scale 1-10)	5.2	5.0	4.8	5.5	5.1
5	Discomfort wearing the patch? (mean; scale 1-10)	2.0	2.3	2.5	1.9	2.2
6	Trouble sleeping due to the patch? (mean; scale 1-10)	1.2	1.8	1.6	1.6	1.6
7	Was the patch in the way? (mean; scale 1-10)	2.7	2.3	2.6	1.6	2.3
8	Did you experience skin irritability? (mean; scale 1-10)	1.4	2.3	3.4	2.3	2.4
9	Did the patch stay in place? (mean; scale 1-10)	7.8	8.3	7.7	8.8	8.1
10	Any restrictions in daily activities due to patch? (mean; scale 1-10)	1.1	1.3	1.4	1.3	1.3
11	Do you have the sense of being watched because of the patch? (mean; scale 1-10)	2.1	1.9	1.9	2.1	2.0

Patients’ reports of skin irritation did demonstrate a change over the patch-wearing period (mean of question 8 at baseline: 1.4; day 1: 2.3; day 5: 3.4). In fact, 3 patients withdrew from the study because of skin irritation. One of these dropouts had a case of relatively severe dermatitis with impacted skin integrity, the other 2 had mild discomfort with minor erythema. The mean of question 8 at day 8, however, normalized to 2.3. The dropout because of skin irritation caused a lower completion of PROs at day 8, biasing results. When withdrawals are excluded, the mean of question 8 at day 5 is comparable with that of day 8. This was also the case for prominent differences at question 3, question 5, question 7, and question 9 (corrected mean for withdrawal at day 5: 2.0, 1.7, 2.5, and 9, respectively). In addition to the questionnaire, connectivity issues between the patch and the phone were reported by 2 patients as bothersome (17%, 2/12). For one of the patients, this led to withdrawal from the study.

### Usability

Usability was measured by a survey completed by 2 research nurses. Placement of the patch was reported to be easy (9/10). Initially, connecting the patch to the phone was at times troublesome but not insurmountable. The ability to monitor patients’ vitals in this study was considered moderately pleasant (6.5/10). Research nurses considered data measured by the VitalPatch to be reliable (8/10).

A total of 4 patients (33%, 4/12) voluntarily withdrew from the study (3 because of skin irritation and 1 because of Bluetooth connection issues). Withdrawals because of skin issues were all reported when wearing the second patch. Withdrawals were evenly distributed over all 3 groups. Neither age, disease, therapy, or other demographic factors showed a trend with withdrawal or skin irritation.

## Discussion

### Principal Findings

This feasibility study focused on the wearability and usability of the VitalPatch in the outpatient setting in patients receiving transfusions or infusions. We quantified the user experience among 12 patients wearing the patch for 12 days. Apart from skin irritation related to the patch in 3 patients, there was positive feedback on comfort and usability of the patch, and emphases on limited restrictions in daily activities because of the patch. Nurses reported ease of use and comfort with relying on data measured by the VitalPatch.

The dropout rate was high compared with earlier work by Selvaraj [14], where out of 70 patients wearing the HealthPatch for 50 days, 6 patients withdrew. Selvaraj also revealed skin irritation issues but also found the need to shave chest hair, personal lifestyle choices, frequent travels, and compensation as reasons for withdrawing from the study [14]. The main reason for dropout in our study was skin irritation. Potentially because our study group patients mainly consisted of patients with hematological malignancies, they were burdened so heavily by their disease and treatment that even relatively small inconveniences were enough to withdraw. Another explanation could be that patients needed more guidance in the first week of wearing the patch. Patients who wore the patch for the whole duration of the study, and thus had gained experience with it, reported little to no inconveniences. Small inconveniences, while missing guidance and experience with the patch, could discourage a patient enough to contribute to discontinuation of use.

Commercial use of the VitalPatch has revealed skin irritation in some subjects, which prompted physIQ’s internal review of the VitalPatch and its relationship to skin irritation. It was discovered that the number of skin irritation reports increased (while statistically nonsignificant) after the introduction of Cavilon as a skin barrier. Therefore, Cavilon use has since ceased to be part of the patch placement instructions. Nonetheless, Cavilon use in this study might explain the dermal issues. Furthermore, we found no correlation in demographics, treatment, or other known parameters with skin irritation. Taking into account that the HealthPatch used in Selvaraj’s trial [14] is a precursor of the VitalPatch, it is more plausible that the high dropout rate found in our study is simply caused by chance and the small sample size and not because of the product itself.

### Strengths and Limitations

Sample size is a limitation of this study. Owing to the exploratory nature of the study, no sample size calculations and no statistical analysis have been applied. Another restriction is that dropouts did not complete the PRO survey at day 8. When comparing the mean values of the PRO survey at day 5 and day 8, it appears time improved patients’ experience with the VitalPatch: PRO data at day 8 were clearly more favorable than those of day 5. However, when corrected for the withdrawals, the PRO means at day 5 and 8 remained the same. Thus, no changes in comfort and skin irritation were observed from day 5 onward. Furthermore, the fact that our cohort was predominantly comprised of older white men reduces its generalizability and is a limitation of this study.

### Relationship to Previous Studies

Overall, comfort, wearability, and usability were comparable with the outcome of Selvaraj’s trial, who also described encouraging feedback on patch-type biosensors for continuous home use [14]. Chan et al [15] looked into the accuracy and usability of measurements of the HealthPatch. These appear to be of comparable accuracy with those made by traditional, larger medical devices. They conclude their article noting that it is important that such a device must be easy to use if it is to be widely adopted. Our data demonstrated ease of use for patients and nurses alike.

### Future Perspective

Besides aiding future studies, the VitalPatch has great potential for in- and outpatient health care. For instance, patients undergoing intensive treatment, such as those receiving an autologous stem cell transplantation, might benefit from continuous monitoring through quick registration and management of adverse events. Finally, in time, when enough experience with the VitalPatch has been gained, it may reduce nurses’ workload by replacing frequent vital sign monitoring.

### Conclusions

Among a variety of wearables [16], this report shows the VitalPatch to be an attractive device because of its wearability and low impact on daily activities in patients, therefore making it suitable for implementation in future studies.

## Appendix

Multimedia Appendix 1 Means of responses. Patients responded by indicating on a Likert-type scale whether they agreed (10) or disagreed (1) with the questions (presented in Table 2). A change in skin irritability (Q8) over time is visible.

## Abbreviations

AE: adverse event
CFR: Code of Federal Regulations
IV: intravenous
PRO: patient-reported outcome
