# Supplementary figures and images for: Remote Patient Monitoring in Adults Receiving Transfusion or Infusion for Hematological Disorders Using the VitalPatch and accelerateIQ Monitoring System: Quantitative Feasibility Study
Source: JMIR Hum Factors. 2019 Dec 2;6(4):e15103. doi: 10.2196/15103 (PMC6915430; doi:10.2196/15103)

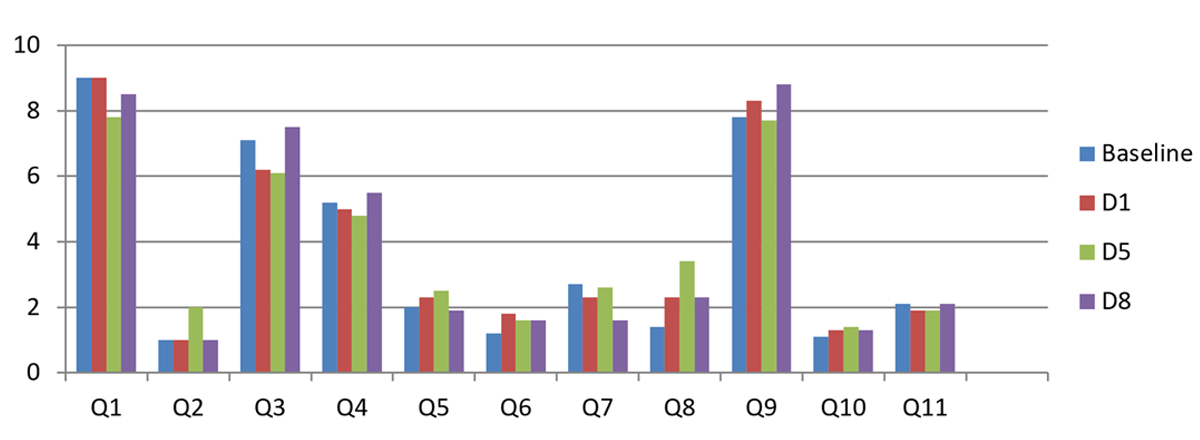

Supplement: Multimedia Appendix 1 [file humanfactors_v6i4e15103_app1.png]
